# Author Correction: Historical changes in wind-driven ocean circulation drive pattern of Pacific warming

**DOI:** 10.1038/s41467-024-48299-w

**Published:** 2024-05-17

**Authors:** Shuo Fu, Shineng Hu, Xiao-Tong Zheng, Kay McMonigal, Sarah Larson, Yiqun Tian

**Affiliations:** 1 Frontier Science Center for Deep Ocean Multispheres and Earth System (FDOMES) and Physical Oceanography Laboratory, Ocean University of China, Qingdao, China; 2 Division of Earth and Climate Sciences, Nicholas School of the Environment, Duke University, Durham, NC USA; 3 Laoshan Laboratory, Qingdao, China; 4 Department of Marine, Earth, and Atmospheric Sciences, North Carolina State University, Raleigh, NC USA; 5 College of Fisheries and Ocean Sciences, University of Alaska Fairbanks, Fairbanks, AK USA

**Keywords:** Climate change, Climate and Earth system modelling, Climate-change impacts

Correction to: *Nature Communications* 10.1038/s41467-024-45677-2, published online 20 February 2024

The original version of this Article contained an error in Eq. (16), where the term $\frac{{\rho }_{s}{c}_{p}H}{\alpha \overline{{LH}}}$ was missing from the ocean advection terms. The correct form of Eq. (16) is:

$${T}_{{advection}}^{t}=	 \frac{{\rho }_{s}{c}_{p}H}{\alpha \overline{{LH}}}\left[{\left(-{u}^{{\prime} }\frac{\partial \bar{T}}{\partial x}\right)}^{t}+\left(-\bar{u}\frac{\partial {T}^{{\prime} }}{\partial x}\right)+{\left(-{u}^{{\prime} }\frac{\partial {T}^{{\prime} }}{\partial x}\right)}^{t}+{\left(-{v}^{{\prime} }\frac{\partial \bar{T}}{\partial y}\right)}^{t}+{\left(-\bar{v}\frac{\partial {T}^{{\prime} }}{\partial y}\right)}^{t}+{\left(-{v}^{{\prime} }\frac{\partial {T}^{{\prime} }}{\partial y}\right)}^{t} \right. \\ 	 \left.+{\left(-{w}_{b}^{{\prime} }\frac{\partial \left(\right.\overline{T-{T}_{b}\left.\right)}}{H}\right)}^{t}+{\left(-\overline{{w}_{b}}\frac{\partial {\left(T-{T}_{b}\right)}^{{\prime} }}{H}\right)}^{t}+{\left(-{w}_{b}^{{\prime} }\frac{\partial {\left(T-{T}_{b}\right)}^{{\prime} }}{H}\right)}^{t}\right]+{{{\rm{R}}}}$$

This has been corrected in both the PDF and HTML versions of the Article.

The original version of this Article contained an error in the Methods, where one term of Eq. (16) was not described. The text in the Methods below equation was updated accordingly to describe this term as follows: “where the overbar denotes the climatological mean state, the prime denotes anomaly, and the superscript ‘*t*’ denotes long-term trend. Each zonal, meridional, and vertical ocean 3-dimensional advection term consists of three components, two linear terms, and one nonlinear term. ${T}_{{advection}}^{t}$ is the sum of the terms on the right-hand side of Eq. (16), excluding the residual terms (*R*), which means that the residual terms are not as important for the heat budget at the mixed layer depth. We use ${T}_{{advection}}^{t}$ for comparison with ${T}_{{Ocn}}^{t}$. *T* is the mixed-layer average temperature, *T*_*b*_ is the bottom of mixed-layer temperature, *u*, *v*, and *w* represent 3D mixed-layer current velocity, *w*_*b*_ is the bottom of mixed-layer temperature and current velocity, and *H* denotes the climatological seasonally varying mixed-layer depth in the equatorial Pacific, which is located at a 0.5 °C difference from the surface SST. *R* is the residual term. The term $\frac{{\rho }_{s}{c}_{p}H}{\alpha \overline{{LH}}}$ varies in space much less than those advective terms. The climatological annual cycle is calculated based on the period from 1958–2014. The anomaly field is calculated by subtracting the monthly mean field from its climatological annual cycle at each period, respectively.

For clarity, the terms on the right-hand side of Eq. (16) are abbreviated in the figures as follows,

17 $$-{u}^{t}\frac{\partial \bar{T}}{\partial x}=\frac{{\rho }_{s}{c}_{p}H}{\alpha \overline{{LH}}}{\left(-{u}^{{\prime} }\frac{\partial \bar{T}}{\partial x}\right)}^{t}$$

18 $$-\bar{u}\frac{\partial {T}^{t}}{\partial x}=\frac{{\rho }_{s}{c}_{p}H}{\alpha \overline{{LH}}}{\left(-\bar{u}\frac{\partial {T}^{{\prime} }}{\partial x}\right)}^{t}$$

19 $$-{u}^{t}\frac{\partial {T}^{t}}{\partial x}=\frac{{\rho }_{s}{c}_{p}H}{\alpha \overline{{LH}}}{\left(-{u}^{{\prime} }\frac{\partial {T}^{{\prime} }}{\partial x}\right)}^{t}$$

20 $$-{v}^{t}\frac{\partial \bar{T}}{\partial y}=\frac{{\rho }_{s}{c}_{p}H}{\alpha \overline{{LH}}}{\left(-{v}^{{\prime} }\frac{\partial \bar{T}}{\partial y}\right)}^{t}$$

21 $$-\bar{v}\frac{\partial {T}^{t}}{\partial y}=\frac{{\rho }_{s}{c}_{p}H}{\alpha \overline{{LH}}}{\left(-\bar{v}\frac{\partial {T}^{{\prime} }}{\partial y}\right)}^{t}$$

22 $$-{v}^{t}\frac{\partial {T}^{t}}{\partial y}=\frac{{\rho }_{s}{c}_{p}H}{\alpha \overline{{LH}}}{\left(-{v}^{{\prime} }\frac{\partial {T}^{{\prime} }}{\partial y}\right)}^{t}$$

23 $$-{w}^{t}\frac{\partial \bar{T}}{\partial z}=\frac{{\rho }_{s}{c}_{p}H}{\alpha \overline{{LH}}}{\left(-{w}_{b}^{{\prime} }\frac{\partial \left(\right.\overline{T-{T}_{b}\left.\right)}}{H}\right)}^{t}$$

24 $$-\bar{w}\frac{\partial {T}^{t}}{\partial z}=\frac{{\rho }_{s}{c}_{p}H}{\alpha \overline{{LH}}}{\left(-\bar{{w}_{b}}\frac{\partial {(T-{T}_{b})}^{{\prime} }}{H}\right)}^{t}$$

25 $$-{w}^{t}\frac{\partial {T}^{t}}{\partial z}=\frac{{\rho }_{s}{c}_{p}H}{\alpha \overline{{LH}}}{\left(-{w}_{b}^{{\prime} }\frac{\partial {(T-{T}_{b})}^{{\prime} }}{H}\right)}^{t}$$

This has been corrected in both the PDF and HTML versions of the Article.

The original version of this Article contained errors in the values shown in panels a, b, d, f of Fig. 3 and Supplementary Fig. 3, resulting from the omission of a term in Eq. (16). In addition, an error appearing in the title of all panels of Supplementary Fig. 4 was corrected from “w_b” to “w”. These have been corrected in both the PDF and HTML versions of the Article.

### Supplementary information

Updated Supplementary Information

