# Supplementary material for: Historical changes in wind-driven ocean circulation drive pattern of Pacific warming
Source: Nat Commun. 2024 Feb 20;15:1562. doi: 10.1038/s41467-024-45677-2 (PMC11258344; doi:10.1038/s41467-024-45677-2)
Supplement: Supplementary file 1 — Supplementary Information [file 41467_2024_45677_MOESM1_ESM.pdf]

Supplementary Information for:

Historical changes in wind-driven ocean circulation drive pattern of Pacific warming

Shuo Fu[1,2], Shineng Hu[2], Xiao-Tong Zheng[1,3], Kay McMonigal[4,5], Sarah Larson[4], Yiqun Tian[2]

[1] Frontier Science Center for Deep Ocean Multispheres and Earth System (FDOMES) and Physical Oceanography Laboratory, Ocean University of China, Qingdao, China

[2] Division of Earth and Climate Sciences, Nicholas School of the Environment, Duke University, Durham, NC, USA

[3] Laoshan Laboratory, Qingdao, China

[4] Department of Marine, Earth, and Atmospheric Sciences, North Carolina State University, Raleigh, NC, USA

[5] College of Fisheries and Ocean Sciences, University of Alaska Fairbanks, Fairbanks, AK, USA

Corresponding author:

Shineng Hu, Division of Earth and Climate Sciences, Nicholas School of the Environment, Duke University. E-mail: shineng.hu@duke.edu

Xiao-Tong Zheng, Frontier Science Center for Deep Ocean Multispheres and Earth System (FDOMES) and Physical Oceanography Laboratory, Ocean University of China. E-mail: zhengxt@ouc.edu.cn

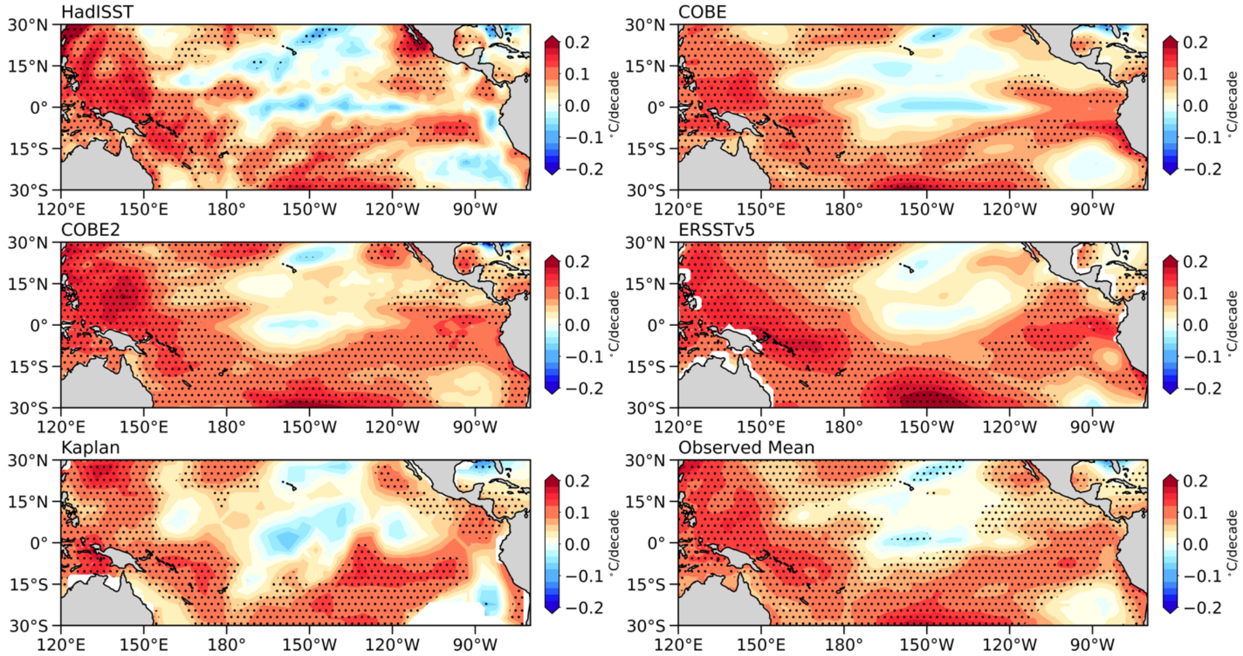

Supplementary Fig. 1 | The sea surface temperature (SST) trend from five observations-based products over 1958–2014. Shown are those from (top left) HadISST, (top right) COBE, (middle left) COBE2, (middle right) ERSSTv5, (bottom left) Kaplan, and (bottom right) the mean of these five observations. Stippling indicates significance at the 95% confidence level.

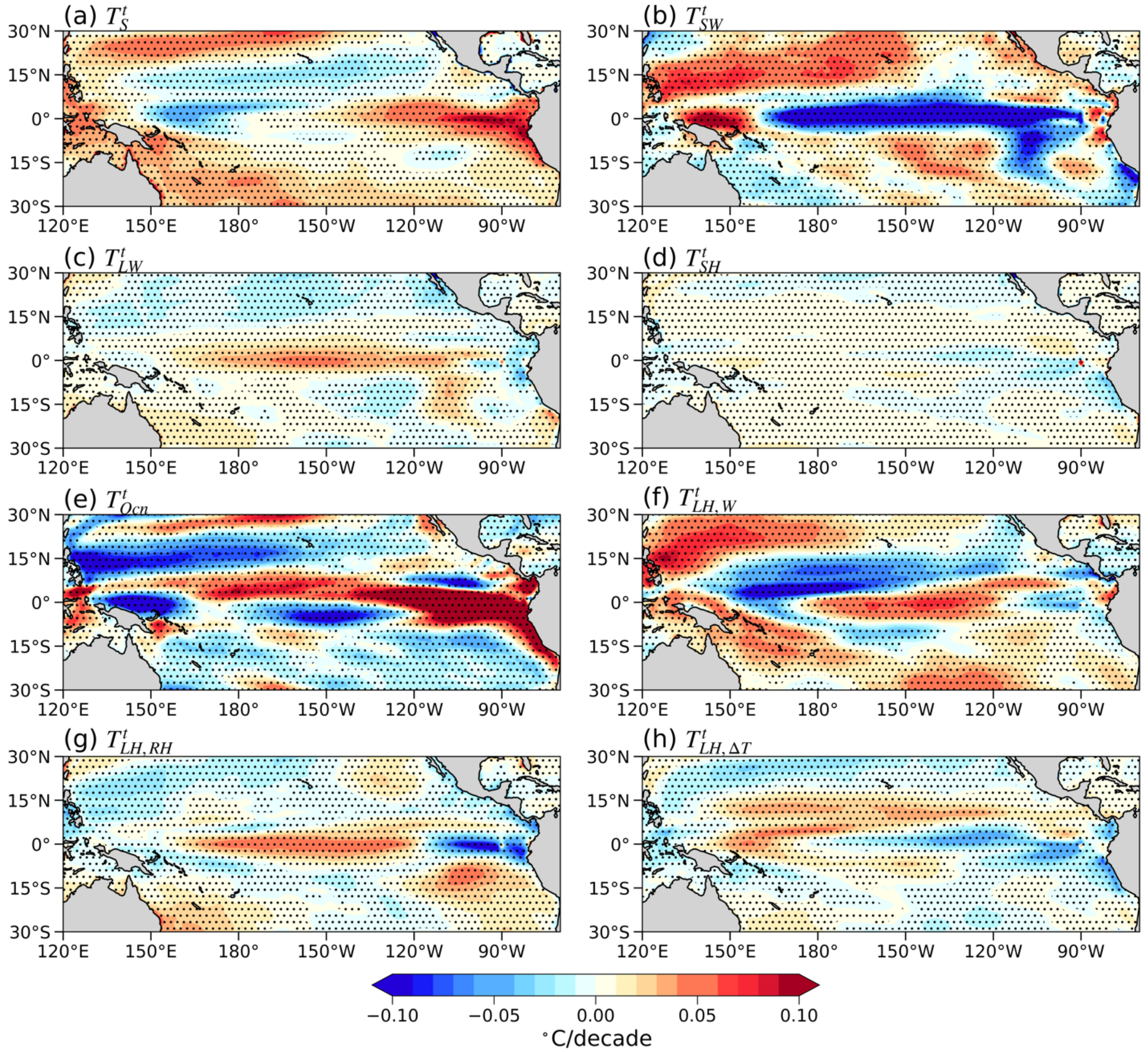

Supplementary Fig. 2 | The decomposition of ocean mixed layer heat budget differences between fully coupled ensemble mean (FC) and mechanically decoupled ensemble mean (MD). a-h, The contribution of FC-MD sea surface temperature (SST) trend from each component in the ocean mixed layer heat budget; see Methods for details. **a,** The sum of all components ($\boldsymbol{T_s^t}$). **b,** Surface shortwave radiative flux trend ($\boldsymbol{T_{SW}^t}$). **c,** Longwave radiative flux trend ($\boldsymbol{T_{LW}^t}$). **d,** Sensible heat flux trend ($\boldsymbol{T_{SH}^t}$). **e,** Heat flux due to ocean dynamics ($\boldsymbol{T_{Ocn}^t}$). **f-h,**

Latent heat flux due to the changes in near-surface wind speed ($\boldsymbol{T_{LH,W}^{t}}$), near-surface relative humidity ($\boldsymbol{T_{LH,RH}^{t}}$), and air-sea temperature difference ($\boldsymbol{T_{LH,\Delta T}^{t}}$), respectively. Stippling indicates significance at the 95% confidence level.

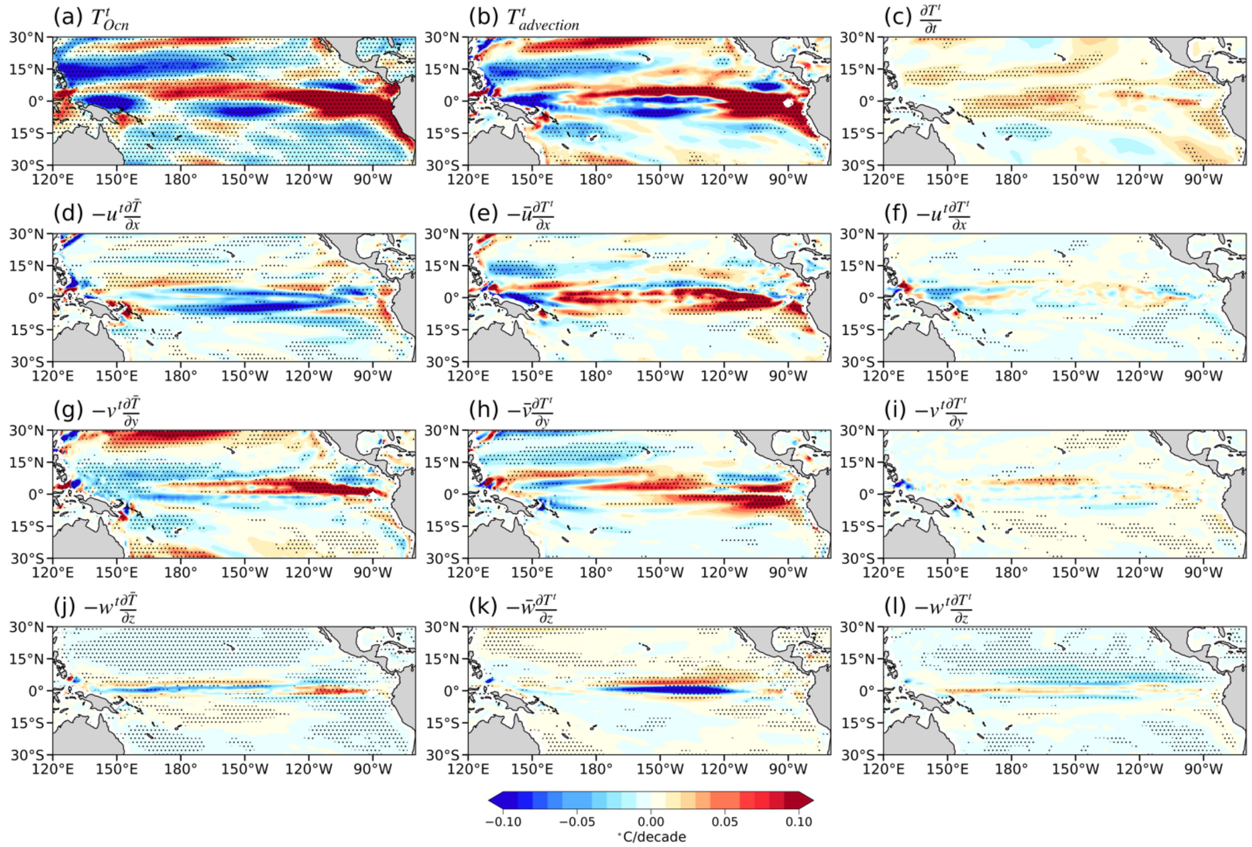

Supplementary Fig.3 | The decomposition of heat budget analysis for ocean dynamical terms between fully coupled ensemble mean (FC) and mechanically decoupled ensemble mean (MD). a, Sea surface temperature (SST) trend due to ocean dynamics (T^t_{ocn}) same as in Fig. 2c. **b,** The total effect of the 9 advective terms ($T^t_{advection}$), **c,** $\frac{\rho_s c_p H}{\alpha \overline{LH}} \frac{\partial (T')^t}{\partial t}$ is the mixed layer temperature tendency, here abbreviated as $\frac{\partial T^t}{\partial t}$, **d-f,** The zonal advective currents terms ($-u^t \frac{\partial \overline{T}}{\partial x}$, $-\overline{u} \frac{\partial T^t}{\partial x}$, $-u^t \frac{\partial T^t}{\partial x}$). **g-i,** The meridional advective currents terms ($-v^t \frac{\partial \overline{T}}{\partial y}$, $-\overline{v} \frac{\partial T^t}{\partial y}$, $-v^t \frac{\partial T^t}{\partial y}$). **j-l,** The vertical currents advective terms ($-w^t \frac{\partial \overline{T}}{\partial z}$, $-\overline{w} \frac{\partial T^t}{\partial z}$, $-w^t \frac{\partial T^t}{\partial z}$). All terms are calculated based on Eq. (16)- Eq. (25); see details in Methods. Stippling indicates significance at the 95% confidence level.

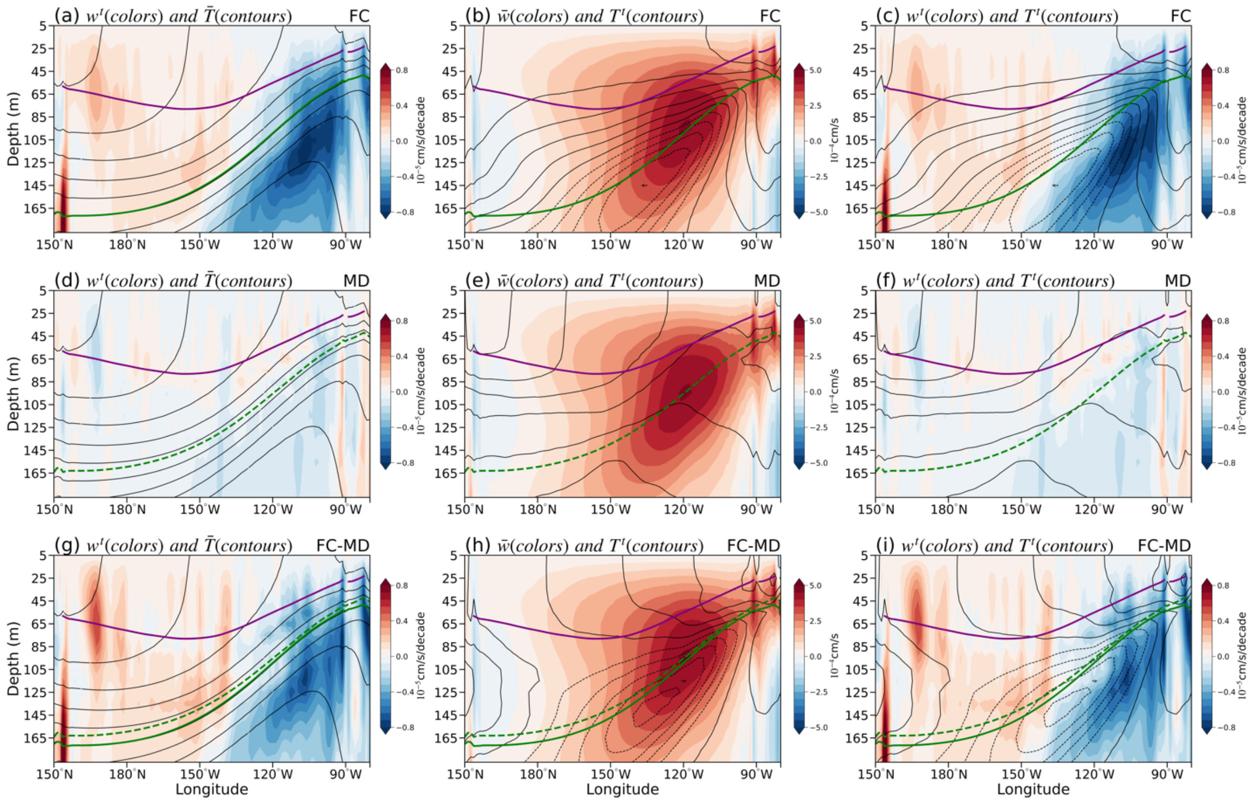

Supplementary Fig.4 | Decomposition of the vertical advection terms in fully coupled ensemble mean (FC), mechanically decoupled ensemble mean (MD), and the difference between FC and MD (FC-MD). The left columns represent the decomposition of the -$w^t\frac{\partial \overline{T}}{\partial z}$ terms in FC, MD, and FC-MD, respectively, the solid line represents the mean state of temperature structure, and the color represents the trend of vertical velocity. The middle columns represent the decomposition of the -$\overline{w}\frac{\partial T^t}{\partial z}$ terms in FC, MD, and FC-MD, respectively, the solid (dashed) line represents the positive (negative) trend of temperature structure, and the color represents the mean state of vertical velocity. The right columns represent the decomposition of the -$w^t\frac{\partial T^t}{\partial z}$ term in FC, MD, and FC-MD, respectively, the solid (dashed) line represents the positive (negative) trend of temperature structure, and the color represents the trend of vertical velocity. The purple line represents the mixed layer depth, and the green solid (dashed) line represents the thermocline depth in FC (MD).

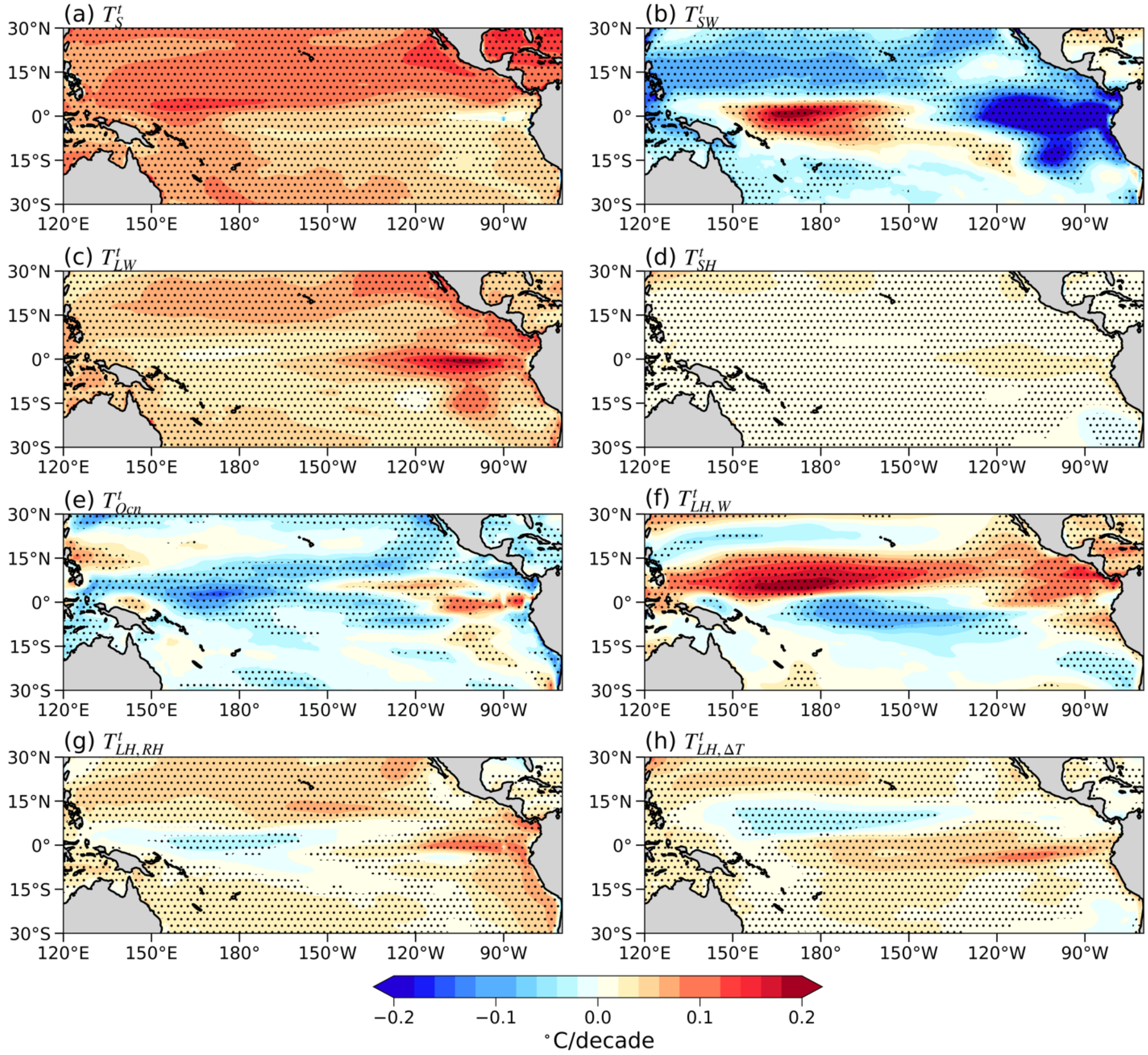

Supplementary Fig.5 | The decomposition of ocean mixed layer heat budget in mechanically decoupled ensemble mean (MD). a-h, MD sea surface temperature (SST) trend decomposition (colors; °C/decade) over 1958–2014 based on Eq. (8). **a,** The sum of all components ($\boldsymbol{T_s^t}$). **b,** Surface shortwave radiative flux trend ($\boldsymbol{T_{SW}^t}$). **c,** Longwave radiative flux trend ($\boldsymbol{T_{LW}^t}$). **d,** Sensible heat flux trend ($\boldsymbol{T_{SH}^t}$). **e,** Heat flux due to ocean dynamics ($\boldsymbol{T_{Ocn}^t}$). **f-h,** Latent heat flux due to the changes

in near-surface wind speed ($\boldsymbol{T^t_{LH,W}}$), near-surface relative humidity ($\boldsymbol{T^t_{LH,RH}}$), and air-sea temperature difference ($\boldsymbol{T^t_{LH,\Delta T}}$), respectively. Stippling indicates significance at the 95% confidence level.

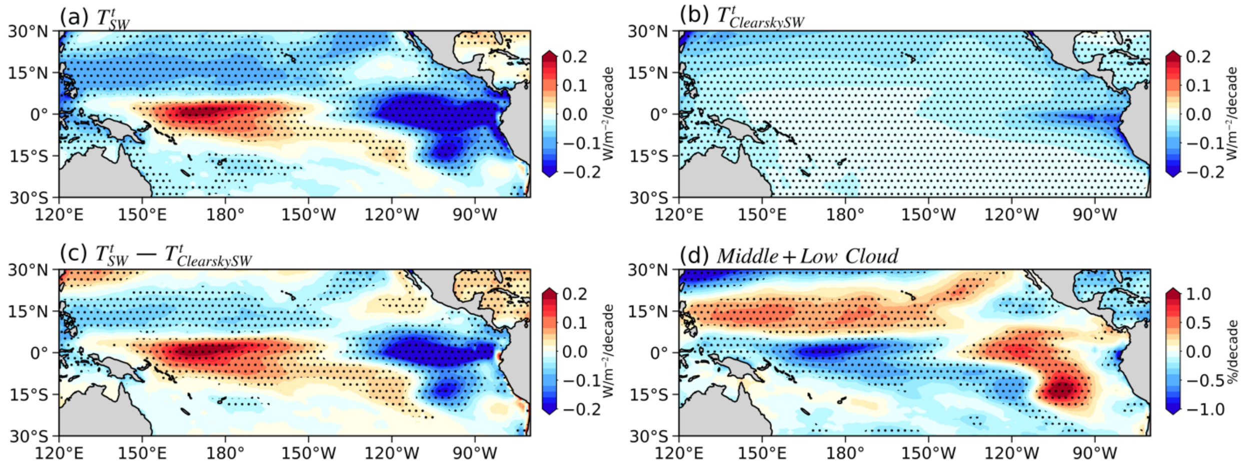

Supplementary Fig.6 | Shortwave and cloud trend in mechanically decoupled ensemble mean (MD). a, The trend of surface shortwave, **b,** the clear sky shortwave, and **c,** the shortwave minus the clear sky shortwave. **d,** The trend of middle and low cloud. Stippling indicates significance at the 95% confidence level.
